# PGE_2_-Induced IDO1 Inhibits the Capacity of Fully Mature DCs to Elicit an *In Vitro* Antileukemic Immune Response

**DOI:** 10.1155/2015/253191

**Published:** 2015-02-26

**Authors:** Sara Trabanelli, Mariangela Lecciso, Valentina Salvestrini, Michele Cavo, Darina Očadlíková, Roberto M. Lemoli, Antonio Curti

**Affiliations:** ^1^Department of Specialistic, Diagnostic and Experimental Medicine, Institute of Hematology “L. & A. Seràgnoli”, University of Bologna, 40138 Bologna, Italy; ^2^Ludwig Center for Cancer Research of the University of Lausanne, 1011 Lausanne, Switzerland; ^3^Department of Internal Medicine (DiMI), University of Genoa and IRCCS Azienda Ospedaliera Universitaria S. Martino-IST, 16132 Genoa, Italy

## Abstract

In the last years, dendritic cells (DC) have been evaluated for antitumor vaccination. Although DC-based vaccines have raised great expectations, their clinical translation has been largely disappointing. For these results, several explanations have been proposed. In particular, the concomitant expression by DCs of tolerogenic pathways, such as the immunosuppressive agent indoleamine 2,3-dioxygenase-1 (IDO1), has been demonstrated. The aim of this study is to evaluate both the stimulatory and the tolerogenic feature of monocyte-derived DCs (Mo-DCs) after maturation with PGE_2_. In particular, the role of IDO1 expression in PGE_2_-matured Mo-DCs has been addressed. Here we show that PGE_2_, which is required for full maturation of DCs, is one mediator of DC tolerance by enhancing IDO1. PGE_2_-mediated expression of IDO1 results in the production of kynurenine, in the generation of T_regs_, and in the inhibition of either the allogeneic or the autologous antigen-specific stimulatory capacity of DCs. When pulsed with leukemic lysates and matured with PGE_2_, DCs are impaired in the induction of IFN-*γ* secreting CD4^+^ and CD8^+^ T cells due to IDO1 upregulation. Moreover, the inhibition of IDO1 enhances the antileukemic response. Overall, these results point toward the use of IDO1 inhibitors to enhance the vaccination capacity of DCs, matured with PGE_2_.

## 1. Introduction

Indoleamine 2,3-dioxygenase-1 (IDO1) is an immunoregulatory enzyme that catalyzes the first and rate-limiting step of tryptophan metabolism along the kynurenine [1]. Its activity is blocked by 1 methyl-tryptophan (1-MT) and, preferentially, by the L-isoform (1-MT-L) [2]. Tryptophan degradation and kynurenine starvation result in the inhibition of T-cell activation, proliferation and survival [3, 4], and in the expansion of regulatory T cells (T_regs_) [5, 6]. The immunosuppressive and tolerogenic role of IDO1 has been observed during maternal tolerance toward the allogeneic fetus [7], regulation of autoimmune disorders [8, 9], and suppression of transplant rejection [10] and in tumor escape [11–15].

Because of their natural features, dendritic cells (DCs) are currently used as cellular vaccines against tumors [16]. In peripheral blood, DCs are a rare population, but the development of protocols to* in vitro* differentiate blood monocytes into DCs (Mo-DCs) triggered the possibility of DC-based immunotherapies [17]. However, DC-based vaccines have demonstrated less clinical efficacy than anticipated. To explain such dismal clinical results, several mechanisms have been proposed. One potential key mechanism is the expression of IDO1 by Mo-DCs. Indeed, Mo-DCs upregulate IDO1 expression upon* in vitro* maturation with the standard cytokine cocktail containing TNF-*α*, IL-1*β*, IL-6, and PGE_2_, used in clinical protocols [18]. It has been shown that PGE_2_ is necessary for the upregulation of CCR7 and metalloproteinase 9 (MMP-9) on DCs and for their consequent migration into lymph nodes [19]. However, PGE_2_ is one of the prominent inducers of IDO1 expression [20]. It has been shown that PGE_2_-mediated IDO1 upregulation in DCs does not impair their capacity of antigen presenting cell [21]. However, IDO1-expressing DCs attract or induce regulatory Foxp3^+^ T cells and the presence of those cells at the site of DC injection suggests possible immunoregulatory effects of IDO1-expressing DCs [22]. Therefore, to improve DC-based vaccine therapy, much attention has to be paid to IDO1 complex modulation by cytokine stimulation of DCs.

Here, we characterize IDO1 expression in human Mo-DCs matured in presence of PGE_2_, in order to test the efficacy of high IDO1-expressing DCs in eliciting an antileukemic response. We found that, combining PGE_2_ with 1-MT-L, it is possible to obtain fully mature Mo-DCs that weakly induce T_regs_ and that elicit IFN-*γ* release by T cells stimulated with acute myeloid leukemia (AML) antigens.

## 2. Materials and Methods

### 2.1. Blood Samples

Cells were obtained from healthy donor buffy coats or from peripheral blood of AML patients who achieved complete remission (CR). PBMC were separated by density gradient centrifugation (Ficoll-Hypaque; Amersham Bioscience, Piscataway, NJ). Cells were cultured in RPMI 1640 medium (Lonza, Milan, Italy) supplemented with 10% heat-inactivated fetal bovine serum (FBS) (Gibco-Invitrogen, Carlsbad, CA, USA), 2 mM L-glutamine, 100 U/mL penicillin, and 100 *μ*g/mL streptomycin (MP Biomedicals, Verona, Italy) at 37°C in 5% CO_2_. CD14^+^, CD3^+^, and CD4^+^CD25^+^ cells were purified by magnetic separation column (Miltenyi Biotec, Bergisch Gladbach, Germany) according to manufacturer's instructions.

### 2.2. DC Generation

Monocyte-derived DCs (Mo-DC) were generated by a 5-day culture of CD14^+^ cells in complete medium supplemented with 50 ng/mL granulocyte-macrophage colony-stimulation factor (GM-CSF) (Endogen, Woburn, MA) and 800 U/mL IL-4 (Endogen), at 37°C in 5% CO_2_ [23]. For maturation, day 5 Mo-DCs were cultured with GM-CSF and IL-4 and incubated for 48 hours in presence of (a) complete medium, (b) 1 *μ*g/mL CD40L (Biolegend, San Diego, CA), (c) 1 *μ*g/mL LPS (Sigma-Aldrich, St. Louise, MO), (d) 1 *μ*g/mL LPS with 100 U/mL IFN-*γ* (Endogen), (e) a cocktail of cytokine made of 10 ng/mL TNF-*α* (Endogen), 10 ng/mL IL-6 (Endogen), 10 ng/mL IL-1*β* (Endogen), with or without 1 *μ*g/mL PGE_2_ (Endogen).

### 2.3. IDO1 Expression

Total RNA was reverse transcribed in 20 *μ*L using Promega Improm II kit and random hexamers (Promega Corporation, Madison, WI, USA). Quantitative real-time PCR (qRT-PCR) was performed using ABI-PRISM 7900 Sequence Detection System (Applied Biosystems, Foster City, CA). qRT-PCR data were analyzed using the 2^−ΔΔCt^ method. The relative level of a specific mRNA for IDO1 was calculated by subtracting Ct values of the control gene (GAPDH) from the Ct values of IDO1. Universal human RNA (Stratagene, Agilent Technologies, Santa Clara, CA) was used as reference and taken as value of 1, IDO1 Assay ID* Hs00158027_m1*, GAPDH Assay ID* Hs00266705_g1*.

### 2.4. DC Immunophenotype

Dual-color immunofluorescence was performed using the following panel of mAbs: PE- or FITC-conjugated anti-human HLA-DR (clone L242, BD Pharmingen); PE- or FITC-conjugated anti-human CD1a (clone HI149, Biolegend); PE- or FITC-conjugated anti-human CD86 (clone IT2.2, Biolegend); PE-conjugated anti-human CD80 (clone 2D10, Biolegend); PE- or FITC-conjugated anti-human CD14 (clone HCD14, Biolegend); FITC-conjugated anti-human CD83 (clone HB15e, Biolegend); FITC-conjugated anti-human CD40 (clone HB14, Biolegend); PE-conjugated anti-human CCR7 (clone 150503, BD Pharmingen). Negative controls were isotype-matched irrelevant mAbs (BD Pharmingen, Biolegend). Cells were analyzed by using FACScan equipment or C6 Accuri equipment (Becton Dickinson). A minimum of 10,000 events was collected in list mode on FACScan software.

### 2.5. Enzyme Activity of IDO1

The amount of L-kynurenine in culture supernatants was measured with a spectrophotometric analysis [24]. Briefly, Mo-DCs were washed, resuspended in Hanks buffer supplemented with 500 *μ*M L-tryptophan (Sigma-Aldrich), and incubated. Supernatants were harvested after 4 h and mixed with 30% trichloroacetic acid (2 : 1), vortexed, and centrifugated at 8000 g for 5 min. Subsequently, this solution was added to Ehrlich's reagent (1 : 1, Sigma-Aldrich) in a 96-well plate. Triplicate samples were run against a standard curve of defined kynurenine concentrations (0–100 *μ*M; Sigma-Aldrich). Optical density was measured at 490 nm, using a Multiskan EX microplate reader (Thermo Electron Corporation, Vantaa, Finland).

### 2.6. Allogeneic and Autologous T-Cell Proliferation

Irradiated (3000 cGy) Mo-DCs were incubated for 5 days with CFSE-labeled CD3^+^ T cells (1 : 10), with or without 1-MT-L (1 mM, Sigma-Aldrich). For autologous mixed leukocyte reaction (MLR) immature Mo-DCs were previously pulsed for 24 h with tetanus toxin (1 *μ*g/mL; Sigma-Aldrich), then washed, and matured. At the end of the culture time, cultures were analyzed using BD FACSCanto II equipment (BD Biosciences).

### 2.7. Induction of Allogeneic and Autologous T_regs_ by Mo-DCs

Mo-DCs were cultured with CD3^+^ T cells (1 : 50) for 5 days with or without 1-MT-L (1 mM). At the end of culture time, cells (1 × 10^5^ cells/100 *μ*L) were incubated with FITC-conjugated anti-human CD4 (clone RPA-T4) and APC-conjugated anti-human CD25 (clone BC96, Biolegend) in the dark for 20 min at 4°C and then for 30 min at room temperature with PE-conjugated anti-human Foxp3 (clone 206D), after 20 min of fixation and 15 min of permeabilization. Samples were analysed using BD FACSCanto II equipment (BD Biosciences). A minimum of 10,000 events was collected in list mode on FACSDiva software.

To test their suppressive activity, at the end of coculture, CD4^+^CD25^+^ T cells (10^4^/well) were purified, irradiated, and added to cultures consisting of CFSE-labeled CD3^+^ T cells (10^5^/well) as responders, stimulated by allogeneic immature Mo-DCs (1 : 10). After 5 days, cultures were analyzed using BD FACSCanto II equipment (BD Biosciences) [25].

### 2.8. Generation of Leukemic Lysate and DC Pulsing

AML cells were resolved in complete medium at 10^7^ cells/mL. Cells were treated with three cycles of heating (10 min at 37°C) and freezing (10 min at −80°C) and the necrotic cell material was filtered throughout a 29G syringe. The cell suspension was added to immature Mo-DCs (2 : 1). After an overnight incubation, pulsed DCs were washed and matured with the cytokine cocktail with or without PGE_2_ [26].

### 2.9. Evaluation of IFN-*γ* Producing CD3^+^ T Cells

Leukemia-reactive IFN-*γ* producing CD3^+^ T cells were evaluated after the coculture with DCs pulsed with leukemic lysate and matured with the cytokine cocktail containing PGE_2_. After 4 h of incubation, brefeldin A was added (2 *μ*g/mL, Sigma-Aldrich) and incubated overnight at 37°C. At the end of the incubation, cell-surface staining was performed as described above (anti-CD4 FITC: clone OKT4, anti-CD8 APC: clone HIT8a, Biolegend). Then, cells were fixed (30 min at 4°C in 2% paraformaldehyde (Sigma-Aldrich)) and the anti-IFN-*γ* antibody (clone B27; Biolegend) was added in 0.1% saponin and incubated for 30 min at 4°C.

Both assays were performed in the presence or absence of 1-MT-L (1 mM). At the end of the culture time, cultures were analyzed using BD FACSCanto II equipment (BD Biosciences).

### 2.10. Statistical Analysis

Results are expressed as mean ± SEM. Depending on experimental conditions analysis has been performed with statistical Student's *t*-test or ANOVA (post hoc Bonferroni), ^*^ *P* < 0.05, ^**^ *P* < 0.01.

## 3. Results

### 3.1. PGE_2_ Enhances IDO1 Expression and Activity

**Figure 1:**
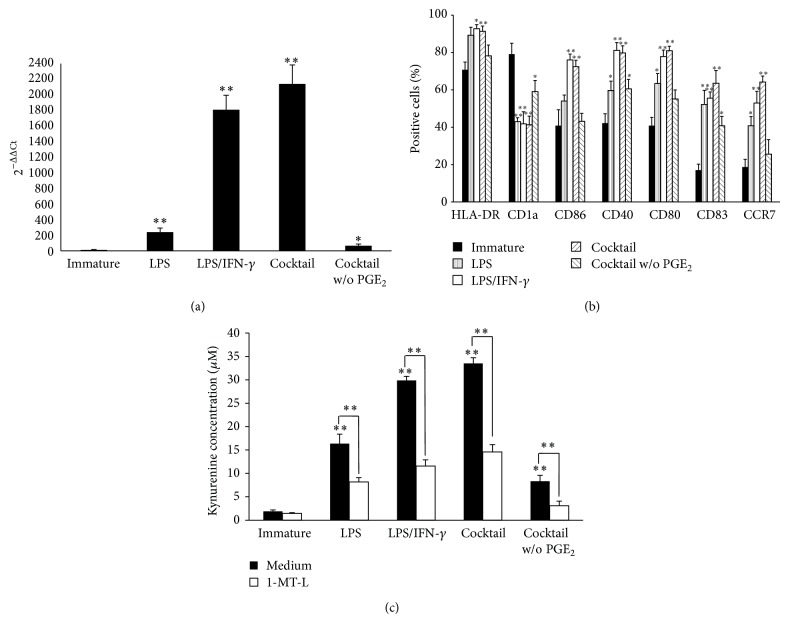
Mature DCs expressed a functionally active IDO1 protein. Human CD14^+^ cells were isolated from healthy donors' PBMC by using magnetic beads. Immature Mo-DCs were obtained after a 5-day culture in presence of GM-CFS and IL-4. Mature Mo-DCs were obtained following 2 days of incubation with LPS, LPS plus IFN-*γ*, the cytokine cocktail made of IL-1*β*, IL-6, and TNF-*α*, with or without PGE_2_. (a) Cells were lysed and RNA was extracted. mRNA expression of IDO1 (normalized to GAPDH) was evaluated by real-time RT-PCR. Universal human RNA was used as reference and taken as value of 1. Data are expressed as the mean ± SEM of 7 independent experiments. ^*^ *P* < 0.05, ^**^ *P* < 0.01 versus immature DCs. (b) DC phenotype was evaluated by flow cytometry. (c) DCs were cultured in presence of 500 *μ*M L-tryptophan for 4 h and supernatants were collected. IDO1 enzymatic activity was evaluated with a spectrophotometric analysis as the production of kynurenine in the supernatants. Data are expressed as the mean ± SEM of 5 independent experiments. ^*^ *P* < 0.05, ^**^ *P* < 0.01 versus immature DCs or versus medium alone.

We first investigated whether different inflammatory stimuli affect IDO1 expression by Mo-DCs during maturation. To this end, we evaluated IDO1 expression in human Mo-DCs after maturation with LPS in presence or absence of IFN-*γ*, or with a cocktail of cytokines including IL-1*β*, IL-6, and TNF-*α*, with and without PGE_2_. Immature Mo-DCs were used as control samples. In line with previous reports [20], maturation of DCs resulted in the significant upregulation of IDO1 (Figure 1(a)). IDO1 was strongly induced by LPS plus IFN-*γ* and by the cytokine cocktail containing PGE_2_. In absence of PGE_2_ the cytokine cocktail induced IDO1 at low level (Figure 1(a)). Of note, in presence of either LPS plus IFN-*γ* or the cytokine cocktail completed of PGE_2_, DCs expressed the highest level of CD80, CD86, CD40, CD83, and CCR7, whereas in presence of the cytokine cocktail without PGE_2_, DCs expressed the lowest level of these markers (Figure 1(b)).

To test the enzymatic activity of IDO1, supernatants of immature and mature DCs cultured with tryptophan-enriched medium were analyzed for L-kynurenine production. As shown in Figure 1(c), L-kynurenine production confirmed, at the functional level, mRNA expression results. Indeed, L-kynurenine concentration was increased after maturation, especially in the presence of LPS plus IFN-*γ* or the cytokine cocktail containing PGE_2_, whereas in absence of PGE_2_ it was weakly increased. L-kynurenine increase was inhibited by 1-MT-L, thus suggesting that the L-kynurenine production was due to IDO1 enzymatic activity.

These findings show that IDO1 expression and function are strongly modulated by the environmental cytokine composition and that PGE_2_, which is required for full maturation, may also act as a switch for IDO1 expression and function.

### 3.2. The Inhibition of IDO1 in PGE_2_-Matured DCs Enhances T-Cell Proliferation

**Figure 2:**
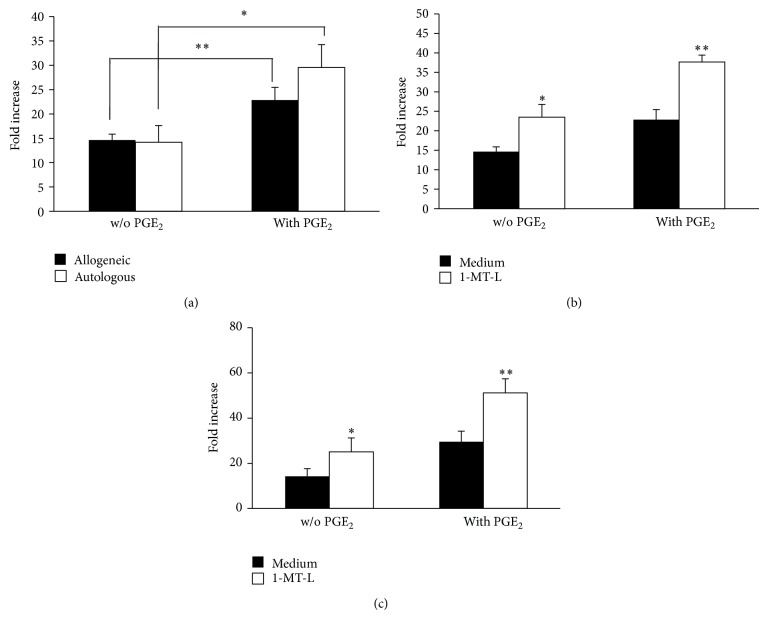
IDO1 expression inhibited the stimulatory capacity of mature DCs. (a) DCs matured with the cytokine cocktail without PGE_2_ (expressing low IDO1) or complete of PGE_2_ (expressing high IDO1) were used as stimulator of either allogeneic or autologous CD3^+^ T cells. For the stimulation of autologous CD3^+^ T cells, DCs were previously pulsed with tetanus toxin. ^*^ *P* < 0.05, ^**^ *P* < 0.01. Both in the allogeneic (b) and in the autologous (c) setting, the assay was performed either in presence or in absence of the IDO1-specific inhibitor 1-MT-L. The results are expressed as the mean of fold increase of 5 independent experiments ± SEM. ^*^ *P* < 0.05, ^**^ *P* < 0.01 versus medium alone.

Once evaluating that PGE_2_ may act as a switch for IDO1 expression, we compared the ability of DCs matured with and without PGE_2_ in inducing T-cell proliferation. We tested whether a high expression of IDO1 (i.e., in presence of PGE_2_) by mature DCs resulted in a stronger inhibition of their ability to stimulate allogeneic or autologous T-cell proliferation in comparison to a low expression of IDO1 (i.e., in absence of PGE_2_). Therefore, we used DCs matured with or without PGE_2_ as stimulators and allogeneic or autologous CD3^+^ T cells as responders. Although PGE_2_ has been shown to confer to DCs some tolerogenic features, such as IL-10 release [27] and IDO1 expression [20, 21], its combination with TNF-*α*, IL-1*β*, and IL-6 enhances their immunogenicity by upregulating costimulatory molecules, stimulating the migration capacity and inducing IL-6 and IL-12 release [28–30]. Indeed, PGE_2_-matured Mo-DCs showed a stronger immunostimulatory activity, both in the allogeneic and autologous setting, than DCs matured without PGE_2_ (Figure 2(a)). However, the addition of 1-MT-L resulted in the significant enhancement of the proliferative capacity of allogeneic (Figure 2(b)) and autologous (Figure 2(c)) T-cells, demonstrating the tolerogenic role of IDO1. Of note, we observed an increase of T-cell proliferation after addition of 1-MT-L also when DCs cultured without PGE_2_ were used as stimulators. Since IDO1 expression in DCs generated in absence of PGE2 is under the level of detection, these results may be explained by an off-target effect of 1-MT-L or by an interference with other tryptophan metabolic pathways, such as tryptophan dioxygenase and/or IDO2.

Taken together, these results suggest that while DCs matured with PGE_2_ retain a stronger capacity of stimulating T-cell proliferation than those matured without PGE_2_, they also upregulate the expression of the immunosuppressive enzyme IDO1. While PGE_2_ is necessary to enhance stimulatory capacity of DCs by inducing a complete maturation, it is also implicated in the induction of tolerogenic pathways, such as IDO1. Indeed, the inhibition of IDO1 further enhances the stimulatory capacity of DCs matured with PGE_2_.

### 3.3. IDO1-Expressing DCs Strongly Induce T_regs_

**Figure 3:**
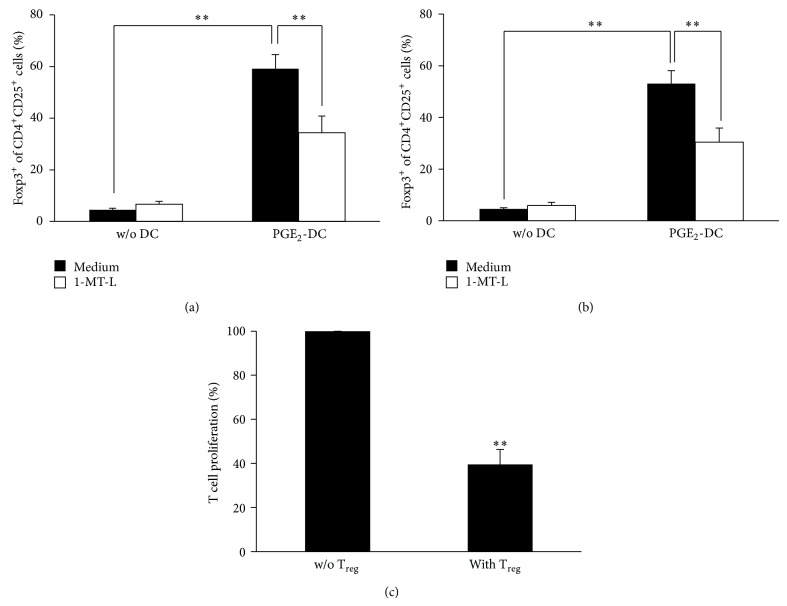
IDO1 expression induced T_regs_. Expression of Foxp3 on (a) allogeneic and (b) autologous CD4^+^CD25^+^ T after 5-day culture with or without high IDO-expressing DCs that is matured with IL-1*β*/IL-6/TNF-*α*/PGE_2_ (complete cocktail), in presence or in absence of the IDO1-specific inhibitor 1-MT-L. The results showed in (a)-(b) are expressed as the mean ± SEM of 5 different experiments. ^**^ *P* < 0.01. (c) CD3^+^ T cells were stimulated by allogeneic immature DCs in the presence and absence of CD4^+^CD25^+^ T cells. CD4^+^CD25^+^ T cells were purified after 5 days of culture in presence of high IDO-expressing DCs (i.e., matured with PGE_2_). The results are expressed as percentage of CD3^+^ T cell proliferation with the proliferation observed in absence of CD4^+^CD25^+^ T cells (w/o T_regs_) as 100%. The results are expressed as the mean ± SEM of 3 different experiments. ^**^ *P* < 0.01 versus w/o T_regs_.

To investigate the role of PGE_2_ in inducing IDO1-mediated T_regs_ generation, we cocultured PGE_2_-matured DCs with purified allogeneic or autologous CD3^+^ T cells. As shown in Figures 3(a) and 3(b), DCs induced a significant increase of the percentage of newly generated allogeneic and autologous CD4^+^CD25^+^Foxp3^+^ T cells, respectively, as compared to CD3^+^ T cells cultured alone. Accordingly, the addition to the cocultures of 1-MT-L significantly reduced the percentage of CD4^+^CD25^+^Foxp3^+^ T cells, thus suggesting that IDO1 represents an important mechanism by which DCs induce CD4^+^CD25^+^Foxp3^+^ T cells in the presence of PGE_2_. To validate their T_regs_ nature, after coculture with PGE_2_-matured Mo-DCs, purified CD4^+^CD25^+^ T cells were able to inhibit T-cell proliferation (Figure 3(c)). Thus, these results show that the CD4^+^CD25^+^ T cells obtained after coculture with IDO1-expressing DCs may be considered* bona fide *T_regs_.

Taken together, these data support the hypothesis that DCs matured in presence of PGE_2_ acquire the ability of inducing a large number of newly differentiated and functional T_regs_ and that such property is linked to the expression of the immunoregulatory enzyme IDO1.

### 3.4. IDO1 Inhibition Enhances the DC Capacity to Elicit the IFN-*γ* Production of Leukemia-Specific T Cells

**Figure 4:**
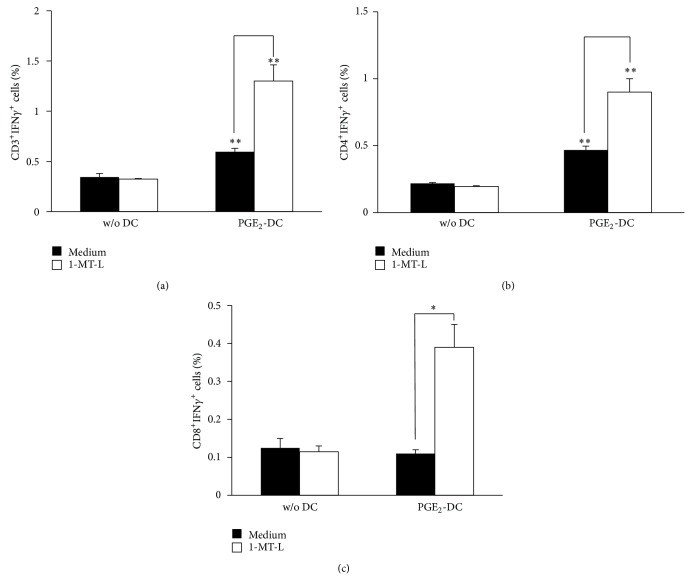
IDO1 expression inhibited leukemia-specific IFN-*γ* production. AML cells were lysed and used for the pulsing of immature Mo-DCs. Mo-DCs were subsequently matured with the cytokine cocktail containing PGE_2_. Autologous CD3^+^ T cells were cocultured with the preloaded and matured DCs for an overnight, in the presence or absence of the IDO1-specific inhibitor 1-MT-L. As control samples, unloaded Mo-DCs were used (data not shown). (a) CD3^+^ T cells, (b) CD4^+^ T cells, and (c) CD8^+^ T cells were tested for leukemia-specific intracellular IFN-*γ* production. Results are expressed as the mean ± SEM of 3 independent experiments. ^*^ *P* < 0.05, ^**^ *P* < 0.01 versus w/o DCs or versus medium alone.

In the attempt to reproduce* in vitro* a model of antileukemia vaccination and to understand the role of PGE_2_ in the induction of an antileukemic response, immature DCs were loaded with necrotic AML blasts, then matured with the cytokine cocktail in presence of PGE_2_, and, finally, used as stimulators for autologous CD3^+^ T cells. As shown in Figure 4(a), Mo-DCs loaded with leukemic antigens were highly efficient in stimulating IFN-*γ*-secreting CD3^+^ T cells. However, although IDO1 inhibition did not impact on the allostimulatory capacity of Mo-DCs previously pulsed with leukemic blasts (data not shown), the addition of IDO1 inhibitor 1-MT-L resulted in a significant enhancement of the percentage of IFN-*γ*-secreting CD3^+^ T cells. This result suggests that IDO1 expression can inhibit the generation of leukemia-specific IFN-*γ*-secreting CD3^+^ T cells. Moreover, among the CD3^+^ T cells, only CD4^+^ T cells were able to produce IFN-*γ* if stimulated with DCs matured with PGE_2_. Of note, the inhibition of IDO1 by 1-MT-L resulted in a significant enhancement of both IFN-*γ*-secreting CD4^+^ and CD8^+^ T cells. Overall, these findings show that the expression of IDO1 in DCs matured in presence of PGE_2_ is able to inhibit the generation of both leukemia-reactive IFN-*γ*-secreting CD4^+^ and CD8^+^ T cells.

### 3.5. PGE_2_ Strongly Upregulates IDO1 Expression in DCs from AML Patients

**Figure 5:**
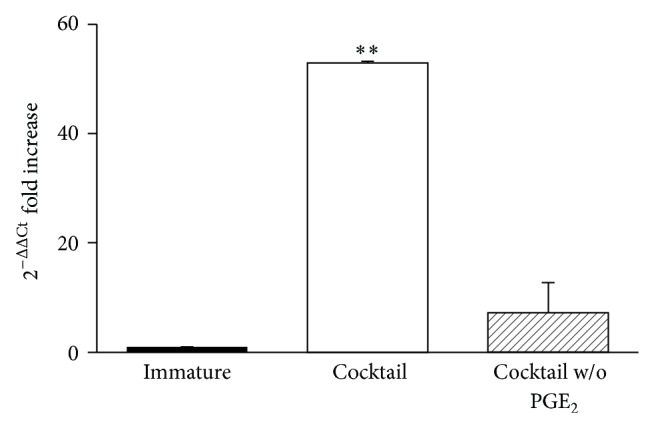
IDO1 expression is enhanced in Mo-DCs from AML patients matured with PGE_2_. Human CD14^+^ cells were isolated from PBMC of AML patients in complete remission by using magnetic beads. Immature Mo-DCs were obtained after a 5-day culture in presence of GM-CSF and IL-4. Mature Mo-DCs were obtained following 2 days of incubation with the cytokine cocktail made of IL-1*β*, IL-6, and TNF-*α*, with or without PGE_2_. Cells were lysed and RNA was extracted. mRNA expression of IDO1 (normalized to GAPDH) was evaluated by real-time RT-PCR. Universal human RNA was used as reference and taken as value of 1. Results are expressed as fold increase of gene expression over immature Mo-DCs (taken as referral condition). The results represent the mean of 3 independent experiments ± SEM. ^**^ *P* < 0.01 versus immature DCs.

To better elucidate the effects of PGE_2_ in the setting of dendritic cell vaccination after chemotherapy-induced remission in acute myeloid leukemia, we evaluated IDO1 expression in normal Mo-DCs obtained from leukemia patients in complete remission. Also in this setting, Mo-DCs were matured in presence of the cytokine cocktail with or without PGE_2_. As expected, in presence of PGE_2_ we found, at mRNA level, a significant upregulation of IDO1 expression (Figure 5). Accordingly with the results obtained with Mo-DCs of healthy donors, we observed the highest expression of costimulation markers in presence of PGE_2_ (data not shown).

These findings confirm that PGE_2_ is a potent inducer of IDO1 expression in DCs from leukemia patients similarly to DCs from healthy donors and might result in the induction of some of the tolerogenic effects observed in DC vaccination which are mediated by the immunosuppressive enzyme IDO1.

## 4. Discussion

Increasing evidence that DC vaccines can induce tumor-specific immune responses in cancer patients is newly leading for the development of therapeutic DC-based cancer vaccines [16, 31, 32]. In this view, it has been shown that type-1 polarized DCs drive Th1-type immune responses that have the potential to mediate tumor therapy through multiple effectors, such as CD8^+^ CTLs and Th1-skewed CD4^+^ T helper cells [33, 34]. Moreover, tumor cell loaded type-1 polarized DCs induce Th1-mediated tumor immunity [35].

In preclinical studies, type-1 polarized DCs are generated, from peripheral blood-derived precursors, by various cocktails of cytokines, commonly including IL-1*β*, TNF-*α*, IL-6, PGE_2_, and/or IFN-*γ* [18]. However, these cocktails of cytokines induce one of the main emerging mechanisms of immunosuppression, IDO1 (present report, [20, 36]), whose expression has been demonstrated to have implications for DC-based vaccines [22]. Notably, the impact on IDO1 expression of the different cytokines which are used for the formulation of DCs-based vaccines is not fully elucidated [37]. In particular the role on IDO1 expression of PGE_2_, which is the most powerful maturation stimulus [38], needs further investigation.

In the present work we firstly compare different maturation stimuli in order to evaluate their capacity in inducing IDO1 and thus in generating tolerogenic Mo-DCs. In agreement with von Bubnoff and colleagues [39], we show that, during maturation, IDO1 expression is enhanced in Mo-DCs. Such result indicates that alongside the induction of full maturation of DCs, inflammatory cytokines and in particular PGE_2_ upregulate IDO1. It has been already demonstrated that PGE_2_ modulates IDO1 expression in circulating DCs from healthy subjects, playing a role in the induction of one of the most important mechanisms of immune tolerance, through the generation of regulatory T cells [38]. Nevertheless, PGE_2_ is known to enhance the ability of DCs to migrate towards the lymph-nodes, as a consequence of upregulation of CCR7 [40]. Furthermore, it has been shown that DCs matured in presence of PGE_2_ retain full ability in inducing allogeneic T-cell proliferation and in stimulating antigen-specific immune responses [28, 29]. In agreement, our data indicate that the presence of PGE_2_ results in an enhanced antigen presenting capacity of DCs. However, our data indicate that PGE_2_ mediates also tolerogenic effect through the induction of IDO1 in Mo-DCs. Indeed, the inhibition of IDO1 with its inhibitor 1-MT-L highly increased the response to tetanus toxin elicited by DCs matured with PGE_2_. Thus, IDO1 expression by human DCs can result in the inhibition of T-cell proliferation (present report and [36]). Furthermore here we show that the addition to the culture of 1-MT-L significantly reduce the percentage of newly generated allogeneic and autologous CD4^+^CD25^+^Foxp3^+^  T_regs_, corroborating the hypothesis that IDO1 overexpression affects the efficiency of the antitumor response. Thus, in the perspective of the development of a DC-based anticancer vaccine, the use of PGE_2_ appears to be essential in order to generate fully mature DCs, but it is also necessary to minimize tolerogenic effects, such as the upregulation of the immunosuppressive enzyme IDO1.

A DCs-based vaccine against leukemia may be a successful strategy for the control or the eradication of the minimal residual disease. We previously demonstrated that in leukemic DCs, generated from acute myeloid leukemia cells, maturation with the complete cocktail of cytokines induces the upregulation of IDO1 gene and protein, thus resulting in tolerogenic effects that have important implications for the use of these cells as vaccines [26]. In the present work, we find that also normal Mo-DCs, obtained from AML patients, show high level of IDO1 expression after maturation with PGE_2_-containing cytokine cocktail. Accordingly, blocking IDO1 activity by 1-MT-L in AML-loaded DCs, matured in presence of PGE_2_ (and consequently expressing high level of IDO1), strongly enhanced the leukemia-specific IFN-*γ* production by T-cells. Our results indicate that an IDO1-mediated immunosuppressive mechanism is involved in weakening the antitumor efficacy elicited by AML-loaded DCs and that specific inhibition of IDO1 might be required for development of cancer vaccines. Our conclusion is in line with recent findings showing that 1-MT enhances the potency of DC-based vaccine against pancreatic adenocarcinoma and Lewis lung carcinoma (LLC) [41, 42]. In fact, in mice, the administration of 1-MT plus DC vaccine caused a slower increase of pancreatic adenocarcinoma as compared to the treatment with either DC or 1-MT alone. In addition, 1-MT enhances the antitumor efficacy elicited by DC/LLC fusion vaccine by delaying the tumor development and inducing stronger splenic CTL responses. Currently, different strategies are under investigation to improve the clinical efficacy of DC-based vaccines for cancer [43, 44]. The use of small interfering RNA to knock down IDO1 expression in a mouse model of breast cancer resulted in the enhancement of the immunogenicity of a DC-based vaccine [45]. Encouraging results were also obtained in cancer patients through immunization with IDO-silenced DCs [46].

## 5. Conclusions

Our paper demonstrates that by combining PGE_2_ with 1-MT-L, it is possible to obtain fully mature Mo-DCs that weakly induce T_regs_ and that elicit IFN-*γ* release by T cells stimulated with AML antigens. Since selective IDO1 inhibitor compounds are currently under clinical investigation, pharmacological IDO1 inhibition is becoming an innovative strategy to potentiate the antitumor efficacy of DC-based vaccines.
